# Dexterity Limitations in Older Adults are on the Rise: Trends in Hand Function Over 20 years and the Effects of Covid-19

**DOI:** 10.1093/geroni/igaf122.2414

**Published:** 2025-12-31

**Authors:** Rachel Logue Cook, Sandra Hunter, Susan Brown

**Affiliations:** University of Michigan, Ann Arbor, Michigan, United States; University of Michigan, Ann Arbor, Michigan, United States; University of Michigan, Ann Arbor, Michigan, United States

## Abstract

Hand dexterity is important for completing activities of daily living and is a strong predictor of functional capacity and independence. However, the prevalence of dexterity limitations among the aging population, particularly after the onset of Covid-19, is largely unknown. We used data from the 2001-2023 cycles of the National Health and Nutrition Examination Survey (NHANES) to identify 15,222 adults aged ≥65. Participants were asked to report whether they had difficulty grasping and handling small objects and were categorized as having a dexterity limitation or no limitation. We determined the population proportion of males and females that reported limitations for each cycle and calculated percentage changes in limitations over the past 20 years and pre- and post-Covid-19. Dexterity limitations remained relatively stable from 2001-2014 for both men (∼16%) and women (∼19%) but significantly increased beginning in 2015 (percentage increase from 2014: 32% males, 26% females) and continued to increase through the 2021-2023 cycle. Before Covid-19 (2017-2018 cycle), 23.6% of males and 26.2% of females reported dexterity limitations and after the onset of Covid-19 (2021-2023 cycle), limitations increased to 25.1% in males(+6.6%) and 34.9% in females(+33.2%), with the greatest increases in adults aged 70-74 (+16%males, +50%females). Hand limitations are increasing among the aging population, particularly women. Covid-19 had detrimental effects on the physical health of many older adults, and our results demonstrated this extended to the hands as well. Understanding the physical limitations experienced by older adults, including the hands, is vital for developing and targeting interventions to maintain health and independence.

